# Metagenomic data-mining reveals enrichment of trimethylamine-N-oxide synthesis in gut microbiome in atrial fibrillation patients

**DOI:** 10.1186/s12864-020-06944-w

**Published:** 2020-07-30

**Authors:** Kun Zuo, Xiaoqing Liu, Pan Wang, Jie Jiao, Chunming Han, Zheng Liu, Xiandong Yin, Jing Li, Xinchun Yang

**Affiliations:** Heart Center & Beijing Key Laboratory of Hypertension, Beijing Chaoyang Hospital, Capital Medical University, 8th Gongtinanlu Rd, Chaoyang District, Beijing, 100020 China

**Keywords:** Gut microbiota, Metagenome, Trimethylamine N-oxide, Atrial fibrillation, Enzyme

## Abstract

**Background:**

The gut bacteria-derived metabolite trimethylamine-N-oxide (TMAO) has been discussed in various cardiometabolic diseases. However, evidence characterizing the microbial population responsible for TMAO accumulation in patients with atrial fibrillation (AF), an increasingly prevalent arrhythmia, is yet lacking. In order to understand the key gut microorganisms that produce TMAO in AF, trimethylamine (TMA)-synthesis enzymes and metabolic pathways, as well as the potential TMA-producers in gut microbiome were assessed based on metagenomic data-mining in a northern Chinese cohort consisting of 50 non-AF controls and 50 patients with different types of AF.

**Results:**

Compared to the control subjects, AF patients showed a marked increase in the microbial genes underlying TMA formation in the gut, which included 12 potential TMA-synthesis functional orthologs and 1 module. The specific bacterial genes, including *choline-TMA lyase*, *carnitine monooxygenase*, *glycine betaine reductase*, and *TMAO reductase*, were elevated in the gut of AF patients. Furthermore, 16 genera were assigned and significantly correlated with TMA-enzymatic genes, where 9 genera were remarkably enriched in the gut communities of AF patients. Neither of these TMA-synthesis pathways nor the microbial players showed a significant discrepancy between different types of AF in the current cohort. These gut microbes might participate in the formation of TMA by activating the key TMA-synthesis enzymes and contributing to the functional pathways in AF patients.

**Conclusions:**

The present study provides an in-depth insight into the potential bacteria and metabolic pathways involved in TMA production in the gut of AF patients. These findings emphasize a key role of the gut bacteria in driving TMAO formation during AF pathogenesis, thereby indicating its therapeutic potential as an intervention strategy of AF by targeting TMA-synthesis pathways and dysbiotic gut microbiota.

## Background

The dysbiosis of gut microbiota (GM) is associated with the pathogenesis of various cardiovascular disorders, such as coronary atherosclerotic heart disease [1–4], chronic heart failure [5, 6], hypertension [7–10], and atrial fibrillation (AF) [11]. Especially, considerable attention has been focused on GM-derived metabolites, such as trimethylamine-*N*-oxide (TMAO). High systemic levels of TMAO heighten the platelet hyperreactivity that increases the risk of thrombosis and elevates the macrophage-specific cholesterol. These features promote atherosclerosis, which in turn, contributes to adverse cardiovascular events, such as acute myocardial infarction, cerebral infarction, and heart failure [12–17]. Several researches have reported the linkage between TMAO and the progression of AF diseases [18–20]. TMAO increases the instability of atrial electrophysiology, induces acute electrical remodeling, and aggravates cardiac fibrosis, thereby facilitating the progression of AF [19, 21]. However, relevant bacterial species and metabolic pathways underlying TMAO production in the gut of AF patients and whether TMAO serves as a pathological link between disordered GM and AF are yet to be elucidated.

TMAO is synthetized through a metaorganismal process, including abundant nutrient precursors in a western diet enriched with choline, phosphatidylcholine and L-carnitine. The gut microbial metabolism of these nutrients results in the production of trimethylamine (TMA), followed by hepatic flavin monooxygenase-dependent conversion into TMAO [15, 22]. Thus, gut TMA formation is a critical process for TMAO accumulation in the host. Several key enzymes involved in the TMA-synthesis pathways have been identified in the gut. For example, the choline-TMA lyase, known as CutC [23], transforms choline into TMA. CntA, an O_2_-dependent carnitine monooxygenase [24], acts on carnitine as wells as its derivative γ-butyrobetaine, leading to TMA synthesis. GrdH [25], is an essential reductase for bacterial degradation of glycine betaine (GBT), and TorA enzyme is a TMAO reductase, which produces TMA with GBT and TMAO, respectively [26]. The gut microorganisms can promote the TMA-production pathways via these key enzymes and utilizing the dietary precursor molecules.

In this study, we performed data-mining of gut metagenomes to assess the shift in the microbial genes with respect to TMA-synthesis enzymes, bacterial functions of potential TMAO-production pathways, and potential microbes harboring these enzymes and functions in AF patients. Thus, the correlation among specific gut microbes, enzymes, and bacterial metabolic pathways key to TMAO production would provide novel insights into the mechanism of GM dysbiosis leading to metabolic disorders via overproduction of TMA in the gut environment of AF patients.

## Results

### Clinical characteristics of the participants

**Fig. 1:**
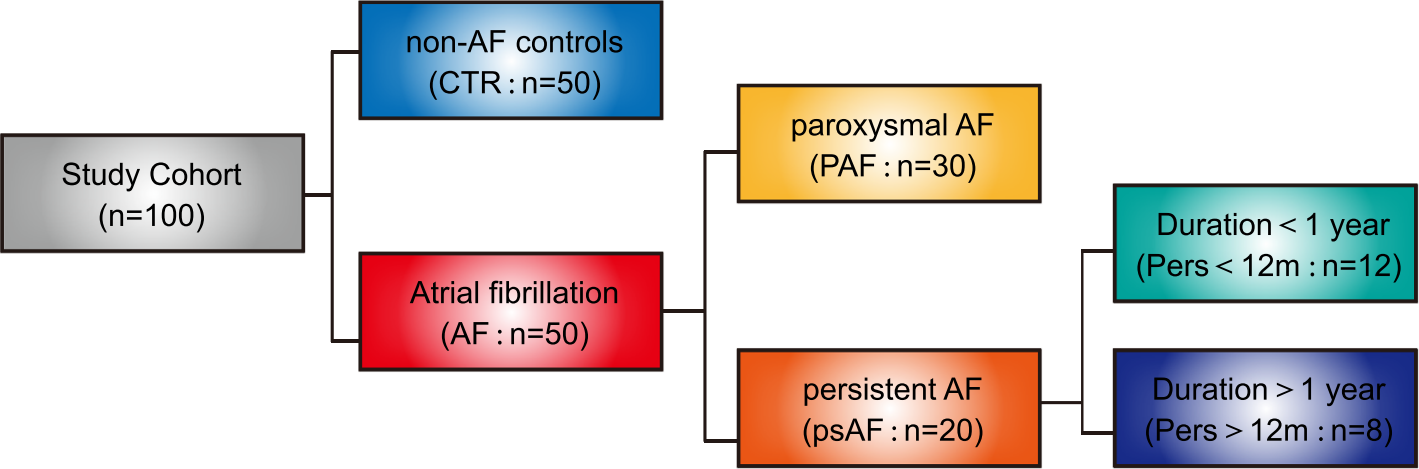
Subject classification. In the present study, 100 individuals were included (patients with AF: *n* = 50; CTR: *n* = 50). Based on the AF duration and character of the electrocardiogram, patients with AF were divided into PAF group (*n* = 30) and psAF group (*n* = 20). The 20 psAF patients were further sub-grouped based on the psAF duration: Pers< 12 m (*n* = 12) and Pers> 12 m (*n* = 8)

In the present study, 100 individuals, aged from 28 to 83 years old, consisting of 50 individuals with AFs and 50 control individuals without AF were included from the previous study [11]. On the basis of AF duration and character of electrocardiogram, the 50 patients with AF were divided into 30 paroxysmal AF (PAF) and 20 persistent AF (psAF). Moreover, the 20 psAF patients were further classified into twelve psAF < 12 months (Pers< 12 m) subjects with psAF duration for shorter than 12 months and eight psAF > 12 months (Pers> 12 m) individuals with psAF duration for more than 12 months. The structure of the current study cohort was described in Fig. 1. The clinical parameters of all the subjects were shown in Supplementary Table S1.

Briefly, patients with AF were more elderly and more complicated by type 2 diabetes mellitus (T2DM) when paralleled with the group of non-AF control (CTR). In our previous study it was found that the age and T2DM have no effect on the present findings in the GM, as individuals of different age and T2DM diagnosis mixed together and were not clustered into divided groups in the principal component analysis scatter plots [11]. The baseline clinical characteristics among the different AF types were similar (PAF vs. psAF; Pers< 12 m vs. Pers> 12 m), with no remarkable difference in terms of age, sex, sex, body mass index, hypertension, T2DM, fasting blood glucose, serum creatinine, or alamine aminotransferase (Supplementary Table S1).

### Functions associated with TMAO-generation in GM of AF patients

The key step in the TMAO production in the host is the conversion of carnitine, glycine betaine, and choline into TMA by GM metabolism [12]. Thus, we focused on the TMA-synthesis functions in the GM of AF patients based on the metagenome data of genes in Kyoto Encyclopedia of Genes and Genomes (KEGG) orthologs and modules. Consequently, a total of 16 TMAO production-related KEGG orthologs and modules were annotated.

Herein, we compared the abundance of TMA-synthesis KEGG orthologs and modules across controls and AF patients to identify the core functions for TMAO generation in AF. Specifically, an odds ratio (OR) score was calculated to measure the differential enrichment of a specific ortholog or module in individuals with AF in comparison to the controls. The ortholog or module with OR score > 2 are considered as being enriched in AF, while with OR score < 0.5 are classified as CTR enriched.

**Fig. 2:**
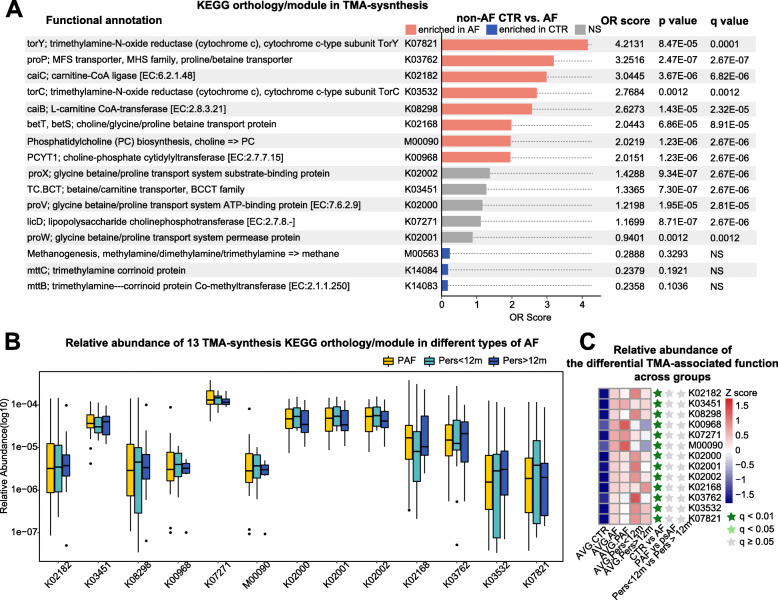
TMA-synthesis of GM is increased in AF patients. **a** The relative abundance of KEGG orthologs and modules associated with TMA-synthesis process in CTR and AF. OR score > 2 (enriched in AF, red), 0.5 < OR score < 2 (not significant, grey), and OR score < 0.5 (enriched in CTR). **b** The box plot shows significantly enriched abundance of 13 KEGG orthologs and modules in the gut of AF patients. Boxes stand for interquartile ranges, with lines representing medians and circles for outliers. **c** Relative abundance of the 13 KEGG orthologs and modules across CTR and AF groups, including PAF, Pers< 12 m, and Pers> 12 m. Statistical difference was defined at the standard of q-value < 0.05 by Wilcoxon rank-sum test, with *P*-value corrected into q-value using the Benjamini-Hochberg method. Abundance profiles after transformation into Z scores through subtracting the mean abundance and divided by the standard deviation. Z scores colored in blue (negative) or red (positive) indicated row abundance less or more than the average, respectively. KEGG orthologs/modules at q-value < 0.01 are shown as an asterisk colored in dark green, q-value < 0.05 with an asterisk colored in light green, and q-value ≥0.05 with an asterisk colored in gray

The majority of the microbial functions for TMA-synthesis were significantly enhanced in the gut of AF patients (Fig. 2a), with seven KEGG orthologs and one module. The microbial genes for carnitine-CoA ligase, betaine/carnitine transporter, L-carnitine CoA-transferase, choline-phosphate cytidylyltransferase, lipopolysaccharide cholinephosphotransferase, glycine betaine/proline transport system ATP-binding protein, glycine betaine/proline transport system substrate-binding protein, choline/glycine/proline betaine transport protein, proline/betaine transporter, trimethylamine-N-oxide reductase, trimethylamine-N-oxide reductase, and phosphatidylcholine PC biosynthesis were elevated in AF individuals. On the other hand, we didn’t identify statistically remarkable difference in the abundance of TMA-synthesis orthologs and modules among different types of AF, including paroxysmal AF (PAF), Pers< 12 m, and Pers> 12 m, which might be limited by small sample size (Fig. 2b, c).

### Increased TMA-synthesis enzymes in AF patients

**Fig. 3:**
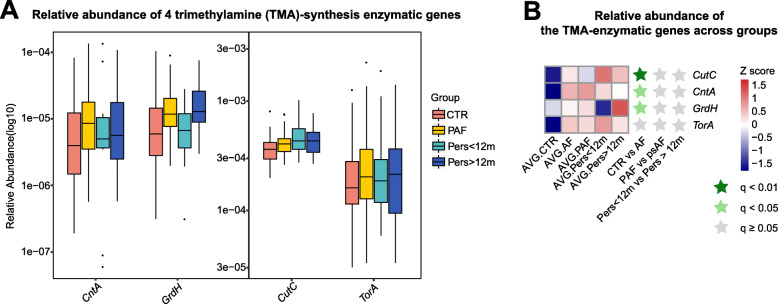
TMA-synthesis enzymatic genes and TMA-associated gut microbes are enriched in AF patients. **a** The box plots show the relative abundance of four TMA-synthesis enzymatic genes in choline, *CutC, CntA, GrdH, and TorA*. Boxes stand for interquartile ranges, with lines representing medians and circles for outliers. **b** Relative abundance of the four TMA-enzymatic genes across CTR and AF groups, including PAF, Pers< 12 m, and Pers> 12 m. Statistical difference was defined at the standard of q-value < 0.05 via Wilcoxon rank-sum test, with P-value corrected into q-value with Benjamini-Hochberg method. Abundance profiles after transformation into Z scores through subtracting the mean abundance and divided by the standard deviation. Z scores colored in blue (negative) or red (positive) indicated row abundance less or more than the average . Genes at q-value < 0.01 are shown as an asterisk colored in dark green, q value < 0.05 with an asterisk colored in light green, and q-value ≥0.05 with an asterisk colored in gray

Additionally, we focused on the specific bacterial genes of enzymes responsible for TMA-synthesis, including *CutC*, *CntA*, *GrdH*, and *TorA*. Intriguingly, *CutC*, *CntA*, and *GrdH* were remarkably upregulated in the gut of patients with AF when compared with non-AF controls (q = 0.0098 for *CutC*, q = 0.0246 for *CntA*, q = 0.0246 for *GrdH*; Fig. 3). Moreover, *TorA* increased in AF, albeit with moderate statistical difference (Fig. 3). Consistent with our findings regarding the microbial functions, the relative abundance of these core TMA-synthesis enzymatic genes was not altered across different types of AF (*CutC*: *P* = 0.3816 for PAF vs. persistent AF (psAF); *P* = 0.9101 for Pers< 12 m vs. Pers> 12 m; *CntA*: *P* = 0.7020 for PAF vs. psAF; *P* = 0.8506 for Pers< 12 m vs. Pers> 12 m; *GrdH*: *P* = 0.5893 for PAF vs. psAF; *P* = 0.0825 for Pers< 12 m vs. Pers> 12 m; *TorA*: *P* = 0.9297 for PAF vs. psAF; *P* = 0.9700 for Pers< 12 m vs. Pers> 12 m; Fig. 3), preliminarily indicating shared profiles of GM in the production of TMA.

### Enrichment of gut bacteria harboring TMA-enzymatic genes in AF patients

**Fig. 4:**
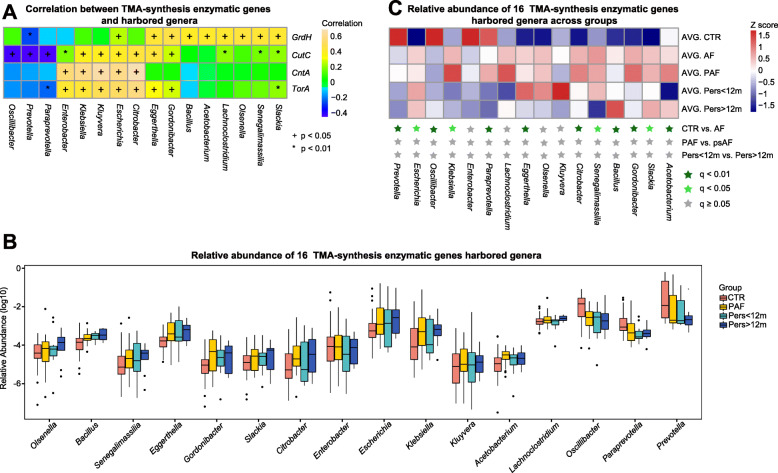
Enrichment of TMA-enzymes harbored by gut bacteria in AF patients. **a**. The relationship between TMA-enzymatic genes and harbored gut bacteria. 16 genera were found to be significantly correlated with TMA-enzymatic genes, *CutC*, *CntA*, *GrdH* and *TorA* (false discovery rate [FDR]-corrected *p* value < 0.05 and correlation at |r| > 0.4). Yellow, positive correlation; blue, negative correlation; +*P* < 0.05, and *P < 0.01. **b**. The box plots show the relative abundance of 16 TMA-enzymatic genes associated genera. Boxes stand for interquartile ranges, with lines representing medians and circles for outliers. **c** Relative abundance of the 16 genera across groups of CTR and different types of AF patients, including PAF, Pers< 12 m, and Pers> 12 m. Statistical difference was defined at the standard of q-value < 0.05 from Wilcoxon rank-sum test, with *P*-value corrected into q-value with Benjamini-Hochberg method. Abundance profiles after transformation into Z scores through subtracting the mean abundance and divided by the standard deviation. Z scores colored in blue (negative) or red (positive) indicated row abundance less or more than the average. The heatmap represents the average of all samples of the bacteria in each group. Taxa at q-value < 0.01 are marked with an asterisk colored in dark green, q-value < 0.05 with an asterisk colored in light green, and q-value ≥0.05 with an asterisk colored in gray

Next, we investigated the gut bacteria harboring TMA-enzymatic genes in AF disease. We aligned the TMA-enzymatic genes, including *CutC*, *CntA*, *GrdH* and *TorA* to the integrated nr database to evaluate the taxonomic allocation. In the current cohort, 98 genera were assigned from TMA-enzymatic genes, where 16 genera significantly correlated with TMA-enzymatic genes (Fig. 4a). Importantly, 4 genera (*Escherichia*, *Klebsiella*, *Kluyvera* and *Citrobacter*) were selected as the taxa strongly associated with TMA-enzymatic genes, where 3 genera of *Escherichia*, *Klebsiella* and *Citrobacter* were remarkably enriched in the gut of AF patients (Fig. 4b, c). For example, *Escherichia* harboring *TorA* was remarkably enriched in AF patients*.* Also, *Klebsiella* harboring *CutC*, *CntA* and *TorA* and *Citrobacter* carrying *CutC* and *TorA* was dominant in AF patients. However, we did not obtain TMA-enzymatic genes associated intestinal bacteria that differed markedly between PAF and psAF (Pers< 12 m and Pers> 12 m) groups (Fig. 4b, c). Our previous study has demonstrated altered GM profiles in AF [11], and based on those findings, we hypothesized that these AF-enriched taxa harboring the TMA-enzymatic genes might be the key microbes contributing to the majority of the TMAO production in the gut of AF patients.

#### Correlation between gut microbes, TMA-synthesis enzymes, bacterial functions, and potential producer of TMAO production in AF

**Fig. 5:**
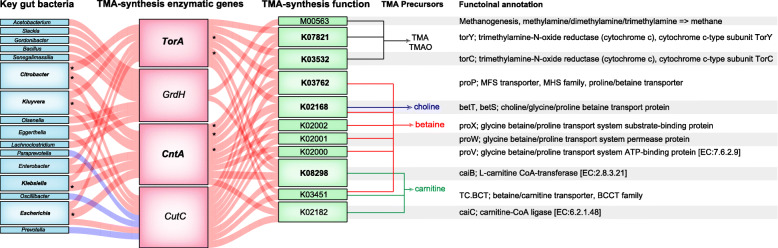
Correlation between gut microbes, TMA-synthesis enzymes, functions and precursors of TMAO production in AF patients. Interrelationship for the TMA-forming potential of microbial communities. Visual presentation of the correlation network based on spearman correlation analysis shows the gut microbes significantly associated with certain TMA-synthesis enzymes, which link to corresponding bacterial functional modules to transform the TMA precursors and participate in the formation of TMAO in AF patients. Red, positive correlation; blue, negative correlation; with statistical significance at FDR corrected *p* < 0.05 and correlation at |r|>0.4, where asterisk marked in boldface means correlation at |r| > 0.6

So as to evaluate the TMA-synthesis potential of bacterial communities in AF subjects, we performed correlation analyses among the gut microbes, TMA-enzymatic genes, and bacterial functions targeting the potential TMA producers. We found that sixteen genera, including *Escherichia*, *Klebsiella, Citrobacter*, and *Kluyvera* were significantly associated with *CutC*, *CntA*, *TorA* or *GrdH* and further correlated with eleven KEGG orthologs / modules (Fig. 5).

The positive network of AF-enriched nine genera (*Escherichia*, *Klebsiella*, *Eggerthella*, *Citrobacter*, *Senegalimassilia*, *Bacillus*, *Gordonibacter*, *Slackia* and *Acetobacterium*) with *CutC*, *CntA*, *TorA* or *GrdH*, might contribute to the activation of bacterial functions, such as carnitine transport, L-carnitine CoA-transferase which participated in the carnitine process, glycine betaine/proline transport system ATP-binding protein, glycine betaine/proline transport system substrate-binding protein, choline/glycine/proline betaine transport protein, proline/betaine transporter that transported glycine betaine, as well as the action of trimethylamine-N-oxide reductase. This complicated correlation might contribute to the metabolism of TMA precursors in the gut of AF patients. These synergistic shifts in the gut microbial composition and function indicated that excessive *Escherichia*, *Klebsiella*, *Kluyvera* and *Citrobacter* colonized in AF patients might participate in the formation of TMAO via TMA-synthesis enzymes, such as CntA and TorA and metabolic functions targeting potential TMA producers.

## Discussion

Although the gut microbe-derived metabolite TMAO contributes to multiple cardiometabolic disorders, including coronary atherosclerotic heart disease, heart insufficiency, and hypertension [12, 13, 16, 18, 27], studies characterizing the fecal bacteria responsible for TMAO production in AF patients are yet lacking. AF is the most prevalent arrhythmia conditions in clinical work, which leads to heavy burden and directly depresses the quality of human life [28–37]. Our previous study characterized the dysbiotic gut microbial features of patients with AF [11]. In the current study, we obtained additional indications depicting the profiles of TMAO-synthesis pathways and potential bacterial producers for TMA in AF subjects based on gut metagenomic data. We also provided a comprehensive understanding of the profiles of TMA-synthesis enzymes. In this respect, bacterial functions contributed to TMA production in the gut environment of AF patients. The potential intestinal bacteria responsible for TMAO formation in AF disease were also identified. Thus, the current findings provided critical clues for further studies on exploring the intervention strategies targeting TMA-synthesis pathways in the gut microbiome to hinder AF progression.

TMAO was previously reported to be associated with concomitant thrombosis risk in patients with elective coronary angiography undergone and also directly influence the platelets in healthy individuals via leading them hyper-responsive, thereby contributing to a prothrombotic phenotype [12, 15, 17, 18]. The underlying mechanisms involve the direct activation of TMAO on the platelets, modifying the release of stimulus-dependent calcium, with increasment in platelet activation with a submaximal level of stimulant, such as thrombin, adenosine diphosphate, or collagen [12]. Other teams have also depicted the similar correlation between elevated TMAO level in heart failure patients as compared to healthy controls [5, 27]. Specifically, an increase in the plasma TMAO level was associated with increased adverse ventricular remodeling, such as fibrosis, chamber enlargement, thinned left ventricular wall, and reduced shortening fraction [27]. Intriguingly, the extent of myocardial fibrosis was relieved when TMAO formation and GM were inhibited by antibiotics [27]. Furthermore, animal data have suggested that increased TMAO levels affect the susceptibility of raised blood pressure [16]. These reports encouraged us to further explore the correlation between the gut bacteria and TMAO production pathways in AF.

One of the major results in the current study was that TMAO-synthesis pathways, including the bacterial genes of TMA-synthesis enzymes, microbial functions, and potential TMA-producers, are abundant in the gut of AF patients. Characterized disturbances of GM in patients with AF, involving elevated GM diversity, specifically altered structure, imbalanced bacterial function, and correlated metabolic profile changes, were depicted by our previous research. Some of the bacteria we discussed here were also found to be differentially represented in our previous study. The TMA-enzymatic genes harbored taxa, such as *Escherichia*, *Klebsiella* and *Citrobacter*, exhibited a remarkable disparity among AF and CTR in this study. These AF-enriched taxa, which harbored TMA-enzymatic genes, might be the key microbes contributing to the maximal metabolic production TMAO in the gut of AF patients. Studies exploring the correlation between high TMAO and cardiovascular diseases support our observations. For instance, *Escherichia* has been reported to be abundant in the gut of coronary atherosclerotic heart disease patients [38], while *Escherichia*, *Klebsiella,* and *Citrobacter* have been identified as genera, containing *CntA* sequences [14]. These previous findings supported the current observations, albeit from another perspective.

A recent study investigating the association between plasma TMAO and occurrence of incident AF showed a significant association between TMAO and subsequent AF in the two cohorts of patients [20]. Although this is an epidemiological study, which did not constitute a cause-effect relationship between TMAO and AF onset, the underlying mechanistic linkage between TMAO and AF has been reported by other investigators. TMAO could enhance the instability of atrial electrophysiology and complicate the substrate of acute electrical remodeling in a rapid atrial pacing-induced AF canine model by aggravating autonomic remodeling [19]. The upregulated levels of inflammatory cytokines in the ganglionated plexi as a result of the activation of p65 NF-κB signaling might facilitate these effects [19]. Furthermore, TMAO exacerbated doxorubicin-induced myocardial fibrosis, at least partially via the activation of the nucleotide-binding oligomerization domain-like receptor protein 3 inflammasome [21]. These epidemiological studies, as well as animal experiments, have demonstrated the crucial role of TMAO in promoting AF development. Therefore, deciphering the state of the TMA-synthesis process in the gut of AF patients is imperative. Herein, we identified that the gut bacterial genes of enzymes responsible for TMA-synthesis, potential gut functions, and key TMA-producers are significantly enhanced in AF patients. Overall, this study provided preliminary evidence about the upregulated TMA-synthesis pathways in the gut of AF patients. Future studies based on a multi-omics approach in a larger cohort may be valuable to demonstrate the correlations between gut bacteria and AF development under clinical conditions.

Nevertheless, the present study has some limitations. First, the discrepancy between different types of AF was not statistically significant, which might be attributed to the sample size of the current cohort. Thus, further studies, including data from other AF cohorts to validate our findings, are essential to substantiate the current observations. Second, the targeted metabolomic analysis was not performed due to the complexity of the sample collection. Hence, we could not obtain the absolute abundance of TMA or TMAO. However, the current findings provide rudimentary evidences for future researches as regards to the possible mechanisms underlying gut bacteria, TMAO, and AF.

## Conclusions

The current study describes the TMAO-synthesis profiles in the gut of AF patients based on the taxonomic composition and bacterial functions. We identified the key gut bacteria responsible for TMAO formation in AF patients. Intervention strategies targeting these detected TMA-synthesis pathways in the gut might be clinically valuable in the future to delay AF progression.

## Methods

### Study cohort

Fifty patients with nonvalvular AF and 50 non-AF controls from northern China were included from our previous study [11]. Individuals with previous heart insufficiency, coronary artery disease, structural cardiac disease, concurrent pathologies such as irritable bowel syndrome, autoimmune diseases, liver or kidney dysfunction, tumor, antibiotic or probiotic applied less than 1 month before recruitment were excluded. The patients of AF were classified into two groups according to AF duration and character of electrocardiogram. PAF is featured with self-termination, in majority less than 48 h and some AF paroxysms may last for up to 7 days, while psAF is regarded as AF that sustains more than 7 days. Moreover, psAF is further classified into two clinically defined groups: 1) Pers< 12 m means a duration of AF for greater than 7 days, but within 1 year; 2) Pers> 12 m means a duration of AF longer than 1 year [28]. The research protocol has been submitted to and approved by the ethics committee of Beijing Chaoyang Hospital and Kailuan General Hospital. All of the individuals enrolled in the current study have signed the informed consent.

### Analyses of metagenomic data-mining

The metagenomic shotgun sequencing data of the 100 fecal samples used in the present study were acquired from a previous study [11]. Fresh fecal samples were obtained from each participant and reserved at − 80 °C. Then, the bacterial DNA was extracted with the TIANamp Stool DNA Kit (DP328, TIANGEN Biotech Co., Ltd., Beijing, China). All samples were processed as paired-end whole-metagenomic shotgun sequencing on the Illumina Novaseq 6000 platform with insert size of 300 bp and read length of 150 bp at Novogene Bioinformatics Technology Co., Ltd. (Beijing, China). The bioinformatic analysis procedure including gene catalog construction, gene prediction, taxonomic annotation and abundance calculation are described previously [11]. Briefly, SOAP2 was applied to realign the reads to the gene catalog by settings of −m 200 − × 400 − s 119. Genes with only ≥2 mapped reads would be involved. The abundance of gene was calculated via counting the number of reads and normalizing by gene length. A total of 612.84 Gb high-quality sequencing reads were obtained (with average 6.13 ± 0.96 million reads per sample).

DIAMOND (version 0.7.9.58, default parameters with the exception of –k 50 –sensitive –e 0.00001) was applied to align all genes in the catalog to the KEGG database (Release 73.1, with genes of animal or plant removed). KEGG is a database resource for functions and utilities of the biological system, which could be categorized into different levels as KEGG orthologs and KEGG modules. KEGG orthologs target metabolism, genetic information processing, environmental information processing, cellular process, organismal systems, human diseases, and Brite hierarchy. The contents of KEGG modules include pathway modules (carbohydrate metabolism, energy metabolism, lipid metabolism, nucleotide metabolism, amino acid metabolism, glycan metabolism, metabolism of cofactors and vitamins, biosynthesis of terpenoids and polyketides, biosynthesis of other secondary metabolites, and xenobiotics biodegradation) and signature modules (gene set and module set), as mentioned on the KEGG platform (https://www.kegg.jp). Each protein would be assigned to KEGG though the highest scoring annotated hits including ≥1 high-scoring segment pair scoring > 60 hits [39]. To identify KEGG orthologs/modules that are associated with AF, the abundance of each ortholog/module in the group of non-AF controls (CTR) was compared to that in the AF group. For each KEGG ortholog/module, k, the odds ratio (OR) was calculated as follows: OR(k) = [∑s = CTR Ask / ∑s = CTR (∑i ≠ k Asi)] / [∑s = AF Ask / ∑s = AF (∑i ≠ k Asi)], where Ask denotes the abundance of KEGG ortholog/module k in sample s. The structure of the formula could be summarized as [sum(s) / sum(a)], where the “sum(s)” denotes the sum of KO1 in AF/CTR group, and the “sum(a)” means the sum of non-KO1 in AF/CTR group. The KEGG orthologs/modules were further classified as AF-enriched (OR > 2) or CTR-enriched (OR < 0.5) [40].

The abundance of genes annotated to the same feature was summed to calculate the abundance of KEGG ortholog/module. The protein sequences of CutC, CntA, GrdH, and TorA were downloaded from the KEGG database. The non-redundant gene catalog was aligned to these sequences through BLASTP (version 2.6.0, best-hit with E-value <1E-5, identity > 50% and coverage > 50%) [41].

To evaluate the taxonomic assignment, the DIAMOND (Version 0.7.9.58, default parameters with the exception of −k 50 − sensitive −e 0.00001) was applied to aligne the TMA-specific genes to the integrated nr database. The valid matches for each gene, regarded as e-values ≤10 × e-value of the top hit, were selected and the reserved matches were performed as previously reported to distinguish taxonomic groups [42]. The lowest common ancestor−based algorithm implemented with MEGAN (MEtaGenome ANalyzer) was applied to determine the taxonomical level of each gene. The abundance of a taxonomic group was calculated by aggregating the abundance of genes annotated to a feature.

### Statistical analysis

Normal distribution quantitative variables were shown as mean ± standard deviation, and compared via the t-test. Non-normal distributed data were expressed as median and quartiles, and analyzed by the Wilcoxon rank sum test. Qualitative variables were interpreted as percentage, with the χ2 test for comparisons. Abundance disparities of genes, genera, and KEGG ortholog/module, were matched by the Wilcoxon rank sum test, with Benjamin and Hochberg correction. Spearman correlation analyses between TMA-related bacteria, enzymes and KEGG ortholog/module were applied by R, and the visualization for Sankey was carried out by R package riverplot (Version 0.6). Two-sided *p* value < 0.05 denoted statistical difference.

## Supplementary information

**Additional file 1 Supplementary Table S1.** Baseline clinical characteristics of the study cohort.

## Data Availability

The data set holding the results of this study has been uploaded to the European Molecular Biology Laboratory (EMBL) European Nucleotide Archive (ENA) with the BioProject accession code PRJEB28384. All the data described here are available at [https://www.ebi.ac.uk/ena/data/view/PRJEB28384].

## Abbreviations

TMAO: Trimethylamine-N-oxide
AF: Atrial fibrillation
TMA: Trimethylamine
GM: Gut microbiota
GBT: Glycine betaine
CTR: Control
PAF: Paroxysmal AF
psAF: Persistent AF
Pers< 12 m: PsAF< 12 months
Pers> 12 m: PsAF > 12 months
T2DM: Type 2 diabetes mellitus
OR: Odds ratio
CutC: Choline-TMA lyase
CntA: Carnitine monooxygenase
GrdH: GBT reductase
TorA: TMAO reductase
KEGG: Kyoto Encyclopedia of Genes and Genomes
ENA: European Nucleotide Archive
FDR: False discovery rate
