# Supplementary material for: Metagenomic data-mining reveals enrichment of trimethylamine-N-oxide synthesis in gut microbiome in atrial fibrillation patients
Source: BMC Genomics. 2020 Jul 30;21:526. doi: 10.1186/s12864-020-06944-w (PMC7391570; doi:10.1186/s12864-020-06944-w)
Supplement: Supplementary file 1 — Additional file 1 Supplementary Table S1. Baseline clinical characteristics of the study cohort. [file 12864_2020_6944_MOESM1_ESM.docx]

|  | **CTR vs. AF** | | | **PAF vs. psAF** | | | **pers<12m vs. pers>12m** | | |
| --- | --- | --- | --- | --- | --- | --- | --- | --- | --- |
|  | **Non-AF CTR**  **(n=50)** | **AF**  **(n=50)** | **P value** | **PAF**  **(n=30)** | **psAF**  **(n=20)** | **P value** | **Pers<12m**  **(n=12)** | **Pers>12m**  **(n=8)** | **P value** |
| **Age, years** | 55  (50.5, 57.5) | 66  (57, 71.25) | <0.001 | 64  (51.75, 70) | 68  (61.25, 72.75) | 0.184 | 65  (58.5, 72) | 68  (62.75, 71.75) | 0.511 |
| **Male/ Female** | 41/9 | 32/18 | 0.015 | 17/13 | 28/8 | 0.190 | 8/4 | 6/2 | 0.698 |
| **BMI** | 24.77  (22.79, 27.62) | 26.46  (23.79, 28.64) | 0.440 | 25.71  (23.47, 27.83) | 27.34  (24.41, 31.24) | 0.120 | 24.71  (22.85, 30.10) | 28.79  (25.29, 33.16) | 0.123 |
| **HTN** | 27 | 27 | 0.954 | 16 | 11 | 0.909 | 5 | 6 | 0.152 |
| **DM** | 0 | 12 | <0.001 | 7 | 5 | 0.894 | 3 | 2 | 1.000 |
| **FBG** | 5.12  (4.56, 5.55) | 4.95  (4.50, 5.83) | 0.541 | 4.95  (4.54, 5.79) | 4.96  (4.47, 5.86) | 0.859 | 5.14  (4.00, 6.48) | 4.70  (4.47, 5.27) | 0.440 |
| **Creatinine** | 70  (60, 89.5) | 68.5  (60.48, 79.35) | 0.218 | 63.8  (59.45, 82.78) | 71.85  (64.95, 77.6) | 0.148 | 69.15  (64.05, 76.95) | 70.45  (61.58, 76.53) | 1.000 |
| **ALT** | 19  (12, 25) | 19  (13.75, 28.5) | 0.191 | 19  (16, 31.25) | 21  (12.25, 28) | 0.728 | 21  (12, 28) | 24.5  (12.25, 44.25) | 0.642 |

**Table S1. Baseline clinical characteristics of the study cohort.**

Abbreviations: CTR, control;AF, atrial fibrillation; PAF, paroxysmal atrial fibrillation; psAF, persistent atrial fibrillation; Pers<12m, persistent atrial fibrillation < 12 months; Pers>12m, persistent atrial fibrillation > 12 months;BMI, body mass index; HTN, hypertension; DM, diabetes mellitus; FBG, fasting blood glucose; ALT, glutamic-pyruvic transaminase; IQR, interquartile range;Data are presented as mean± SD, or median (IQR), as appropriate.
